# Definitive radiochemotherapy *versus* surgery within multimodality treatment in stage III non-small cell lung cancer (NSCLC) - a cumulative meta-analysis of the randomized evidence

**DOI:** 10.18632/oncotarget.16471

**Published:** 2017-03-22

**Authors:** Christoph Pöttgen, Wilfried Eberhardt, Georgios Stamatis, Martin Stuschke

**Affiliations:** ^1^ Department of Radiotherapy, West German Cancer Center, University Hospital, University of Duisburg-Essen, Essen, Germany; ^2^ Department of Medical Oncology, Ruhrlandklinik, West German Cancer Center, University Hospital, University of Duisburg-Essen, Essen, Germany; ^3^ Department of Thoracic Surgery, Ruhrlandklinik, University Hospital, University of Duisburg-Essen, Essen, Germany

**Keywords:** meta-analysis, radiochemotherapy, trimodality, NSCLC

## Abstract

Randomized trials were analyzed comparing surgery with definitive radiotherapy as local curative treatment options within the framework of different multimodality treatments for patients with locally advanced non-small cell lung cancer (NSCLC). Endpoints for comparison of treatment results were overall survival, progression-free survival, and toxicity.

Hazard ratios (HR) were taken to measure treatment effects and pooled using a random effects model.

Overall survival was not significantly different between surgical and definitive radiotherapy arms (HR=0.92 [95%CI 0.82-1.04], *p*=0.19, χ^2^-test). There was heterogeneity with respect to survival at 2 years (*p*<0.0001, Cochran Mantel Haenszel (CMH)-test). Latter trials using concurrent radiochemotherapy (ccRT/CT) showed better survival at 2 years (risk ratio of death=0.80 [95%CI 0.73-0.88], *p*<0.0001, CMH-test). In the ccRT/CT trials, survival in the surgical arms tended to have an excess early mortality before 6 months of follow-up and a lesser hazard rate in comparison to definitive ccRT/CT thereafter (HR=0.78 [95%CI 0.63-0.98]). Over all trials, treatment associated mortality was higher in the surgical arms (risk ratio=3.56 [95% CI: 1.65-7.72], *p*=0.0005, CMH test). With respect to progression-free survival, no significant differences were found (HR=0.91 [95%CI: 0.73 - 1.13]), although the largest conducted trial found an advantage for the surgical arm (HR=0.77 [95%CI: 0.62-0.96]).

Induction therapy followed by resection or definitive radiochemotherapy represent valuable curative treatment options for patients with stage III NSCLC, the individual treatment choice deserves careful interdisciplinary evaluation and counseling. Based on the broad heterogeneity of patient groups in these stages further research on predictive factors supporting individual therapy selection is necessary.

## INTRODUCTION

About 25-30% of all non-small cell lung cancer patients are diagnosed primarily with locally advanced cancers representing one of the most heterogeneous tumor groups. Due to several permutations of tumor and lymph node (T and N) descriptor combinations, stage III according to the revised UICC classification (7^th^ edition) includes patients with limited tumor size and single-level mediastinal lymph-node involvement on one hand and bulky infiltrative masses involving major mediastinal structures on the other. Criteria of resectability are difficult to define and thus the use of surgery as such as well as the optimal sequence of treatment modalities is an area of continuing controversy.

The UICC classification is mainly based on outcome of surgical series. Major changes in the current 8^th^ revision include the conversion from T4 N0 (former IIIB) tumors into stage IIIA considering the obvious prognostic improvement after resection in contrast to other IIIB tumors. [1] The same applies for multiple lung lesions which have been converted from M1 to either T3 (within the same lobe) or T4 (within the same lung) (8^th^ revision). In contrast to these changes, mediastinal lymph node involvement has remained unchanged and the attribution to stage IIIA or IIIB as well. Further sub-classifications of the mediastinal involvement have been suggested by Robinson and the College of the American Chest Physicians, which partly represent criteria of potential resectability. [2, 3]

Following current guidelines, concurrent radiochemotherapy represents the standard of care in patients with locally more advanced tumors (without malignant pleural effusion) while in potentially resectable stage IIIA tumors bimodal or trimodal treatment regimes including surgery represent alternative standard options. [4, 5]

Here we summarize the best current evidence comparing definitive radiotherapy or radiochemotherapy and multimodality treatment including surgery within randomized trials.

## RESULTS

Seven studies with randomization have been identified (Table 1). [6–12] One of these has been presented as abstract only [11] while all others have been published as full papers. Due to relevant treatment cross-over, one study of these [7] was excluded from further analysis (only 4 patients resected in the surgical arm, compliance < 20%). Six studies including 1322 patients were evaluated for final metaanalysis. These studies cover treatment periods through two decades (1990's until 2012, Table 1) and reflect the differences of diagnostic investigations (CT, PET, PET/CT) and treatment over time with a variety of combinations of first-, second- and third-generation drugs as well as the change from older radiation techniques towards three-dimensional conformal radiation therapy. Only two studies reached almost full accrual (EORTC, INT 0139). [9, 10] The remaining trials were closed earlier due to evolving evidence for the superiority of concurrent radiochemotherapy (RTOG 89-01, NCIC, NTOG) [7, 8, 11] or slow accrual (ESPATUE) [12], respectively.

**Table 1:** Randomized studies comparing induction treatment followed by surgery with definitive radio(chemo)therapy

Trial (Period of Recruitment)	Inclusion Criteria	Treatment	median OS [mo]	long-term OS	Hazard Ratio	*P*
**RTOG 89-01** (1990-1994)	IIIA N2	[R]* (1) 2x CDDP/VBL (MMC) → S (2) 2x CDDP/VBL (MMC) → RT [64 Gy]	19.4 17.4	22.0% [4Y] 22.0%	n.g.	0.46
**NCI Canada** (closed 1995)	IIIA N2	[R] (1) 2x CDDP/VBL → S (2) ---------------------------- → RT [60 Gy]	18.7 16.2	n.g.	n.g.	NS
**MRC** (1995-1999)	IIIA	[R] (1) 4x CDDP/MMC/IFO or VBL → S (2) ----------------------------------------------- → RT [40-60 Gy]	13.8 11.2	n.g.	0.91 [0.49-1.72]	0.78
**EORTC 08941** (1994-2002)	IIIA N2	(1) 3x CDDP/ 3rd gen drug → **[R]°** → S [+PORT 56 Gy] (2) 3x CDDP/ 3rd gen drug → RT [60-62.5 Gy]	16.4 17.5	15.7% [5Y] 14.0%	1.06 [0.84-1.35]	0.596
**Nordic TOG** (1998-2009)	IIIA N2	[R] (1) 3x carboplatin/paclitaxel → S [+PORT 60 Gy] (2) 3x carboplatin/paclitaxel → RT [60 Gy]	17.3 14.9	19.0% [5Y] 17.0%	0.866	0.218
**INT 0139** (1994-2001)	IIIA N2	[R] (1) 2x CDDP/ETOII45 Gy/1.8 Gy qd → S → 2x CDDP/ETO (2) 2x CDDP/ETOII45 Gy/1.8 Gy qd → RT [61 Gy]→2x CDDP/ETO	23.6 22.2	27.0% [5Y] 20.0%	0.87 [0.7-1.1]	0.24
**ESPATUE** (2004-2012)	IIIA N2 selected IIIB	(1) 3x CDDP/paclitaxel♢ CDDP/VINII45 Gy (AHF)♢[R] → S (2) 3x CDDP/paclitaxel♢ CDDP/VINII45 Gy (AHF) → RT [20-26 Gy♢65-71 Gy] +CDDP/VIN	49.3 34.8	44.0% [5Y] 40.0%	0.81 [0.5-1.3]	0.34

Abbreviations: [R]: randomisation timepoint, [R]*: only 45 of 73 patients randomised, [R]°: only responders randomised, OS: overall survival, (1): arm 1 – induction treatment plus surgery, (2): arm 2 – conservative treatment: combined radio(chemo)therapy without resection, mo: months, --: no chemotherapy, S: surgery, RT: radiotherapy, CDDP: cisplatin, VBL: vinblastin, IFO: ifosfamide, MMC: mitomycin, ETO: etoposide, VIN: vinorelbine, n.g.: not given, NS: not significant, PORT: postoperative radiotherapy, qd: once daily, II: concurrent, AHF: accelerated hyperfractionation.

Patient eligibility criteria comprised (potentially) resectable non-small cell lung cancer stage III. In four of the studies, resection after chemotherapy alone was planned in the surgical arm [6, 8, 9, 11], while in two others induction radiochemotherapy (45 Gy) followed by resection was foreseen. [10, 12]

In general, radiotherapy was given with conventional fractionation. [6, 8–10] Hyperfractionated-accelerated radiotherapy was used during induction radiochemotherapy in ESPATUE (45 Gy, 1.5 Gy twice daily) and as an alternative regimen to conventional fractionation in the NTOG trial (61.2 Gy, 1.7 Gy twice daily). [11, 12]

Induction chemotherapy was mainly based on platinum-based doublets, in older trials in combination with vinblastine, later combined with second or third generation agents (etoposide, gemcitabine, paclitaxel). Except INT 0139 and ESPATUE, radiotherapy was administered as sequential modality after induction chemotherapy. Within the Intergroup trial, patients received cisplatin/etoposide simultaneously with 45 Gy (1.8 Gy/fraction) whereas Eberhardt et al. [12] have used cisplatin/vinorelbine during the concurrent radiochemotherapy phase.

Randomization was planned either before treatment or after induction CT or RT/CT (Table 1). In the latter trials, responding patients, or patients with resectable tumors after induction were randomized. [9, 12] Due to a midterm revision of the RTOG trial protocol, only 45 of 73 eligible patients have been randomized in that study. [8]

Specifications for radiotherapy as well as surgical procedures per protocol were developed continuously and increasingly detailed throughout the accrual time. Protocol modifications based on refinements of inclusion criteria or quality control, respectively, were made in RTOG 89-01, EORTC 08941, and ESPATUE.

Surgical procedures are presented in detail in Table 2. A considerable shift from pneumonectomy to more lung-tissue-sparing resection techniques can be observed over time. While the amount of pneumonectomies accounts for 47% of resections in the EORTC trial, a steady decrease in the number of pneumonectomies is present in INT 0139 and ESPATUE with an amount of 35% and 33%, respectively.

**Table 2:** Surgical procedures and results (only treatment arm: induction plus surgery)

Study (Period of Recruitment)	Resection			Pathologic Response [% of resected]	Downstaging [% of resected]
	Lobectomy	Pneumonectomy	Complete Resection / Resected	pCR	MediastinalNodalClearance
**RTOG 89-01** (1990-1994)	23		19/26	n.g.	n.g.
**NCI Canada** (closed 1995)	n.g.	n.g.	10/13	0 [0]	n.g.
**MRC** (1995-1999)	1(1 SleeveResection)	2	4/4	n.g.	n.g.
**EORTC 08941** (1994-2002)	59	72	77/154	8 [5]	39 [25]
**NordicTOG** (1998-2009)	n.g.	n.g.	121/132	n.g.	45 [34]
***INT 0139*** *(1994-2001)*	*98*	*54*	*155/155*	*29* [19]	*47* [30]
***ESPATUE*** *(2004-2012)*	*46*	*23*	*66/70*	*21* [30]	*38* [54]

n.g. - not given, trials using simultaneous chemotherapy during induction are given in italics.

In addition, it is noteworthy that the rates of mediastinal nodal clearance and pathologic complete remissions have steadily increased during the last decade and have reached an amount of 61% and 30% of resection specimen, respectively, in ESPATUE (Table 2), which is statistically significantly higher than in the trials before (*p* < 0.001, χ^2^-test).

### Overall survival

**Figure 1:**
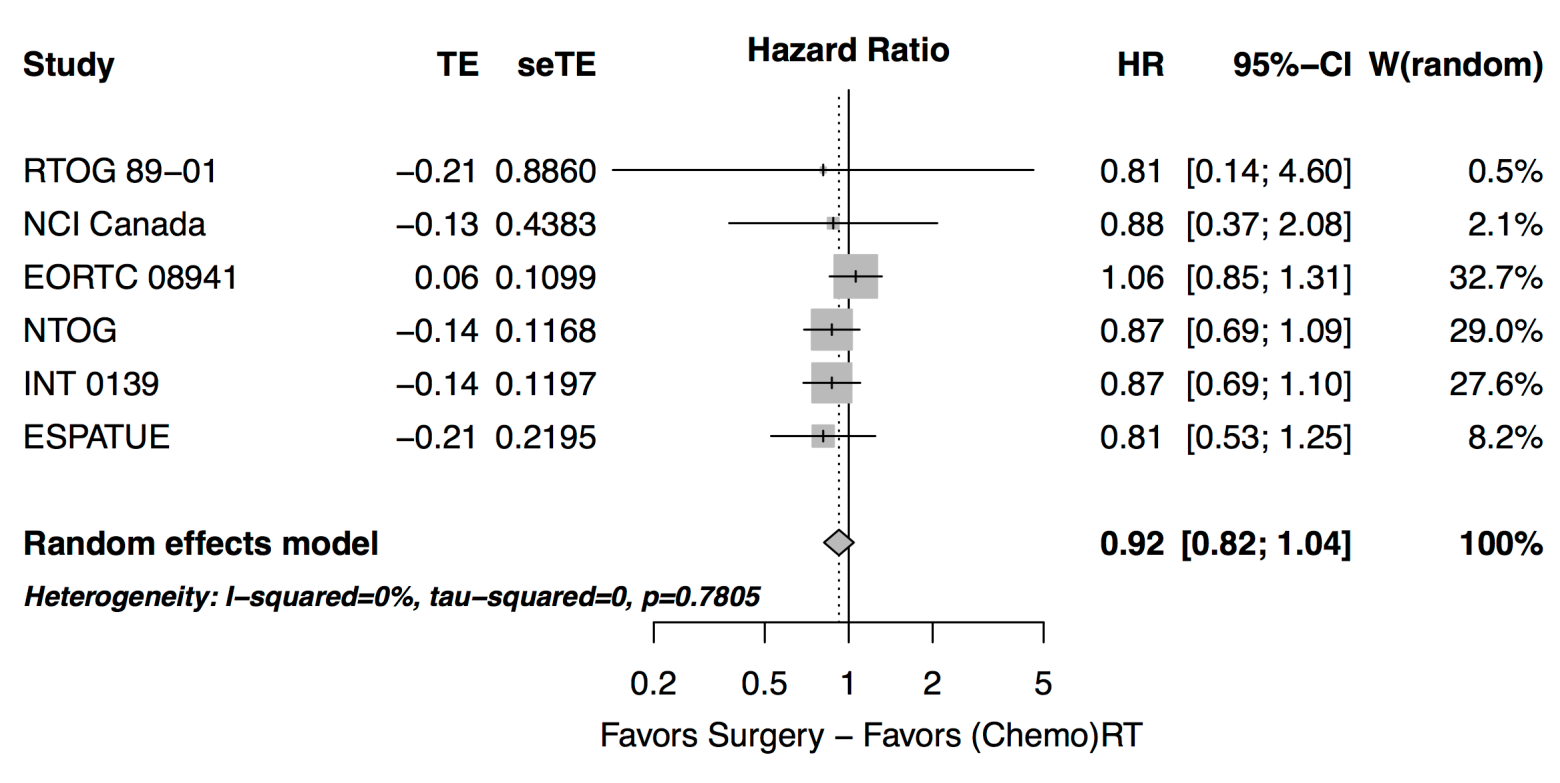
Forest plot: overall survival - randomized prospective studies, experimental: treatment arm with surgery

Median survival durations in the studies with a recruitment period ending before 2000 range from 11 to 19 months while in the later trials a steady increase in long-term survival duration was achieved. A comparable evolution of 5-year survival rates between 14% and 44% after randomization was noted (Table 1). No overall survival difference between treatment arms was detected when comparing a surgical arm with definitive radiochemotherapy using the intent to treat data from all six trials (hazard ratio 0.92 [95% CI 0.82-1.04], *p* = 0.19, χ^2^ test, Figure 1). No significant hetereogeneity between the hazard ratios of the trials was detected (τ^2^ = 0.0, Q = 2.47, *p* = 0.78).

There was heterogeneity across studies in overall survival at two years (*p* < 0.0001, Cochran Mantel Haenszel (CMH)-test). For this analysis, all patients initially recruited to the trial and not only the randomized patients were counted in the case of the ESPATUE trial. When comparing the risk of death within two years between the trials using concurrent radiochemotherapy [10, 12] and those trials which used sequential chemo-radiotherapy [8, 9, 11] or radiotherapy alone [6] in the non-surgical arm, the risk ratio was 0.80 (0.73-0.88) favouring simultaneous radiochemotherapy (*p* < 0.0001, CMH test). Considering the subgroup of trials using concurrent radiochemotherapy separately, again no significant overall effect was observed between arms (hazard ratio 0.86 [0.70 - 1.05], *p* = 0.14). No significant hetereogeneity in the respective hazard ratios was detected between the latter trials (τ^2^ = 0.0, Q = 0.08, *p* = 0.78).

However, qualitative analysis revealed that survival curves of both treatment arms crossed at an early time point of follow-up between 12-18 months in the INT 0139 and ESPATUE trial. An exploratory analysis revealed that the hazard ratio of death between arms in the stratified analysis of the ESPATUE and the Intergroup trial was 1.53 (95% CI: 0.95- 2.47) considering the time period between 0 - 6 months of follow-up alone. The hazard ratio in patients who have survived 6 months (left truncation at 6 months) was 0.78 (95%CI: 0.63-0.98). The hazard ratio between the surgical arm and the radiochemotherapy arm was increased in the early time period ( < 6 months) by a hazard ratio of 1.95 (95%CI: 1.16-3.31, *p* = 0.013) in comparison to the time period of > 6 months of follow-up. For this detailed analysis, individual times to death or end of follow up were used from the ESPATUE trial and the respective distribution of failure times or censoring events were obtained from the digitized figure from the publication of the INT 0139 trial [10]. This is, in principle a deviation from the proportional hazard assumption, but this did not become significant by a Kolmogorov-type maximum supremum-test (*p* = 0.11).

### Progression-free survival

**Figure 2:**
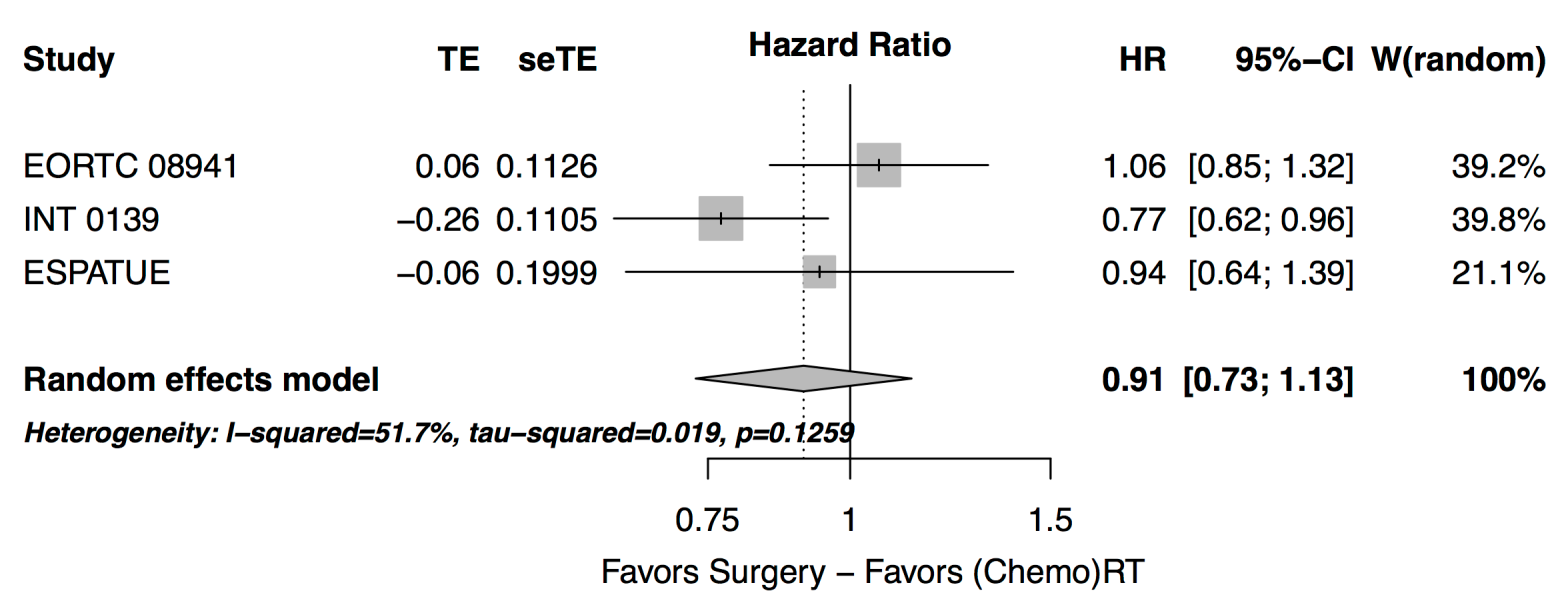
Forest plot: progression-free survival - randomized prospective studies with published progression-free survival rates, experimental: treatment arm with surgery

Data on progression are limited and given in a subset of the studies only (Table 2). In the three trials reporting progression-free survival rates, no overall difference between treatment arms was observed (hazard ratio 0.91 [0.73 - 1.13], *p* = 0.4, Figure 2). Especially, the significant benefit in terms of progression-free survival in favor of the surgical arm (HR = 0.77 [95%CI: 0.62-0.96]) from the INT 0139 trial was not found in the EORTC trial (HR = 1.06 [95%CI: 0.85-1.33]) and the ESPATUE trial (HR = 0.94 [95%CI: 0.63 - 1.39]). The heterogeneitiy of the hazard ratios from these trials did not become significant at the progression-free survival end point (τ^2^ = 0.2, Q = 4.14,*p* = 0.13).

### Treatment related toxicty

Over all trials, a significantly higher odds ratio of treatment-related deaths is found in the surgical arms of the studies (risk ratio = 3.56 [95% CI: 1.65-7.72], *p* = 0.0005, CMH test, Table 3). The cumulative evidence for an excess mortality increased over time and became significant after the EORTC trial. In that study, 47% of the resected patients received a pneumonectomy which, as well as in INT 0139, had a negative influence on survival. Nevertheless, risk ratios were homogeneous among trials (*p* = 0.88, Breslow Day test).

**Table 3:** Results

Study (Period of Recruitment)	Treatment- related Deaths after Randomisation		Postoperative Deaths due to Surgical Complications/Resected	Cancer- related Deaths		Local - Regional Recurrences		Distant Metastases		Number of patients alive at 1 year follow-up		Number of patients alive at 2 years follow-up		Patients per Arm	
	Arm 1	Arm 2	Arm 1	Arm 1	Arm 2	Arm 1	Arm 2	Arm 1	Arm 2	Arm 1	Arm 2	Arm 1	Arm 2	Arm 1	Arm 2
**RTOG 89-01** (1990-1994)	2	1	2/26	n.g.	n.g.	n.g.	n.g.	n.g.	n.g.	19	21	13	11	29	32
**NCI Canada** (closed 1995)	0	0	0/13	11	10	8	6	1	4	12	10	7	6	16	15
**MRC** (1995-1999)	2	0	2/4	16	19	n.g.	n.g.	n.g.	n.g.	13	10	3	3	24	24
**EORTC 08941** (1994-2002)	6	1	6/154	n.g.	n.g.	45	80	78	59	104	115	58	66	167	165
**NordicTOG** (1998-2009)	n.g.	n.g.	n.g./132	n.g.	n.g.	n.g.	n.g.	n.g.	n.g.	107	104	67	60	170	171
***INT 0139*** *(1994-2001)*	*16*	*4*	*15/155*	*109*	*136*	*21*	*43*	*98*	*110*	*136*	*131*	*97*	*89*	*202*	*194*
***ESPATUE*** *(2004-2012)*	*5*	*2*	*5/70*	*31*	*41*	*n.g*.	*n.g*.	*n.g*.	*n.g*.	*63*	*66*	*51*	*47*	*81*	*80*

Arm 1 – induction treatment plus surgery, (2): Arm 2 – conservative treatment: combined chemoradiotherapy without resection,

n. s. – not significant, n.g. - not given, trials using simultaneous chemotherapy within the definitive radiochemotherapy arms are given in italics.

## DISCUSSION

The present metaanalysis including the recently published randomized ESPATUE trial did not found significantly different overall survival in patients with locally advanced NSCLC after induction treatment and surgery compared with those receiving definitive radiochemotherapy. There was a significant variability across trials with respect to overall survival. Trials using concurrent radiochemotherapy in the neoadjuvant phase of the resection arm and simultaneous radiochemotherapy in the other arm showed a better overall survival than the other trials using induction chemotherapy alone before surgery in the surgical arm and sequential chemotherapy and radiotherapy or radiotherapy alone in the other.

Concomitant radiochemotherapy is standard treatment for definitive radiochemotherapy at least since the meta-analysis of Auperin et al. (2010). [14] The observed lack of significant differences in overall survival between treatment arms of the trials comparing a surgical arm with a radiotherapy or radiochemotherapy arm did not change with prognosis and the hazard ratio for survival was similar for the trials using or not using concomitant radiochemotherapy.

That this across studies comparison might to some extent reflect a selection bias cannot be ruled out. Staging methods have been refined over the accrual periods, e.g. PET/CT was consistently used for staging in the recent ESPATUE trial. Surgical as well as radiotherapy procedures have been increasingly well defined. However, quality assurance is a special challenge within the multicentre setting and the difficulties to reach sufficient patient recruitment underline the problems associated with an adequate performance of such an intensive treatment regimen within a prospective trial.

As stage III comprises a heterogeneous group of tumors, selection of patients for resection remains difficult in terms of technical resectability, as well as medical, or prognostic operability. During the enrolment period of the studies analyzed here which spans two decades of research, most trials recruited T1-3 N2 patients alone and the separation between stages IIIA and IIIB has been not clear in some of the trials. From a clinical point of view, this renders a specific comparison of treatment results difficult in different subgroups of stage III patients.

Treatment-related toxicity represents an important factor for the N2 non-small cell lung cancer patient cohort with a negative influence on overall survival. While all cause mortality was similar in the randomized trials, patients in the surgical study arms faced a relevant risk of treatment associated mortality which is mainly attributable to the risk of postoperative death, and overall mortality is significantly higher than after radiotherapy. In general, surgery is feasable and tolerable, even with pneumonectomy, once dedicated multimodality centers become involved. [15] In the EORTC trial, an exploratory subgroup analysis showed a significantly worse outcome for patients who underwent pneumonectomy in comparison to those who underwent (bi-)lobectomy (HR 0.59, 95% CI = 0.40 - 0.87). [9] In INT 0139, an initially unplanned overall survival matching analysis for four prestudy factors for the surgery arm against chemoradiotherapy subsets was prompted by an unexpectedly high mortality rate in the surgical arm mainly associated with pneumonectomies. This analysis was undertaken for 90 of 98 lobectomies and 51 of 54 pneumonectomies. The overall survival rate was improved in the surgical group if a lobectomy was done compared with the rate in the matched chemotherapy plus radiotherapy group (5-year survival 36% *versus* 18%). In contrast to INT 0139, no increased association of mortality with pneumonectomy in ESPATUE was observed. [12] However, an increasing complexity of the surgical intervention is typically associated with an increased risk of postoperative mortality. The trials using concomitant radiochemotherapy showed a cross-over in survival between surgical and non-surgical treatment arms at times between 12 and 18 months. Obviously, during the early follow-up period, hazards of death are somehow increased for patients undergoing surgery, while during later follow-up, advantages for overall survival appear in resected patients.

Explorative analyses of the randomized trials pointed out predictive factors that might have a value for treatment selection, i.e. adenocarcinoma histology, the T1N2 subgroup and primary tumors that can be resected with lobectomy. [10, 11] However, these factors were not prospectively validated and other large studies did not find a negative influence of adenocarcinoma histology on survival after definitive radiochemotherapy or a negative influence of pneumonectomy after neoadjuvant treatment. [15–17]

It has been shown consistently that subgroups of patients who achieve mediastinal nodal clearance or pathological complete remission (pCR), respectively, will have favorable survival, especially after lobectomy. However, good responders to definitive chemoradiotherapy also have an improved survival, as assessed by the standardized uptake decrease on [^18^F]-FDG PET [18]. The predicteve effect of standardized uptake decrease was not found to be different between the treatment arms in that secondary analysis of the ESPATUE trial [18].

Currently, based on the finding of a comparable outcome in survival in the randomized trials, the safer approach of radiochemotherapy remains the preferred approach in many institutions. Surgery may represent a good treatment choice within a multimodality treatment program for patients in good condition and upfront potentially resectable tumors provided that patients will be treated by an expert team incorporating all disciplines of thoracic oncology ensuring a high level of expertise.

## MATERIALS AND METHODS

### Data sources, study selection, Data extraction and data synthesis

PubMED^®^, Medline^®^ and Web of Science^®^ have been searched to identify randomized studies comparing definitive local treatment (radiotherapy, radiochemotherapy, trimodality treatment) in patients with locally advanced stage III non-small cell lung cancer. Search criteria were: (lung cancer) AND (random*) AND radiotherapy AND surgery AND (stage III OR stage IIIA OR N2). Primary endpoint was overall survival. Secondary endpoints were progression-free survival, and toxicity. Clinical data (chemotherapy, radiotherapy details, surgical procedures, locoregional recurrences, distant metastases) and survival data (treatment-related and cancer related deaths, postoperative deaths due to complications,) were extracted from the original publication, independent data extraction by multiple observers was performed. In addition, randomized studies that were published between 2000 to 2015 as abstracts on the annual meetings of ASCO and ASTRO were searched according to the aforementioned criteria and analyzed using the full meeting presentation.

Hazard ratios (HR) from Cox proportional hazard analysis were taken as published to measure treatment effects. Survival curves from publications not presenting hazard ratios (e.g. [6]), were fully digitized using currently available public-domain software for image analysis. [19] After cross-check with the survival data from the respective publication, Cox proportional hazard analysis was performed using SAS software, hazard ratios were calculated using proc PHREG (SAS^®^, version 9.4, Cary, NC). Analysis of the proportional hazard assumption was performed by a Kolmogorov-type supremum test for the proportional hazard assumption (proc PHREG, SAS^®^). For publications without complete survival data presentation (e.g. [8]), a general parametric approach was applied using p-values from the log-rank test to derive hazard ratios. [20] Visual inspection showed a crossing of the survival curves for both treatment arms of the INT 0139 and ESPATUE trials at an early time point of follow-up between 12-18 months. To assess this effect, the hazard at early time points < 6 months after randomisation was simultaneously compared with the hazard function at later times > 6 months in both arms of the ESPATÜ trial and the INT 0139 using the PHREG procedure. For this analysis, the authors had full access to the original survival data of the ESPATUE trial, as published in Eberhardt et al. (2015, ESPATUE being an acronym for Essen-Paris-Tuebingen, representing the initially planned study centers). [12]. The respective distribution of failure times or censoring events were obtained from the digitized figure from the publication of the INT0139 trial [10].

Meta-analysis calculation was performed in R using the Meta and Metafor packages. [21, 22] Ratio measures (hazard ratios) have been entered as natural logarithm (effect size) together with the corresponding standard errors from the individual studies and the pooled estimate was calculated from an inverse-variance-weighted average of the individual studies. A random-effects method was applied to account for study effects variability. All reported p-values are two-tailed. Between-study heterogeneity was assessed using τ^2^-statistics. Frequencies of events in both arms were analyzed with proc FREQ (SAS^®^).

Publication bias was examined using funnel plots of the estimated treatment effect for each trial against the respective standard error.

#### Data access, responsibility, and analysis

C. Pöttgen and M. Stuschke had full access to all the data in the study and take responsibility for the integrity of the data and the accuracy of the data analysis.

### Authors' contributions

All authors have substantially contributed to the conception of the present work. Data collection, and statistical analysis have been performed indpendently by Christoph Pöttgen and Martin Stuschke. C.P, M.S., and Wilfried Eberhardt have written the manuscript, Georgios Stamatis has critically revised the manuscript. All authors have approved the final version of the manuscript, and are accountable for all aspects of the work in ensuring that questions related to the accuracy or integrity of any part of the work are appropriately investigated.
